# Polyphenolic Characterization and Anti-Inflammatory Effect of In Vitro Digested Extracts of *Echinacea purpurea* L. Plant Parts in an Inflammatory Model of Human Colon Cells

**DOI:** 10.3390/ijms25031744

**Published:** 2024-02-01

**Authors:** María Ángeles Ávila-Gálvez, Juan Antonio Giménez-Bastida, Bulent Karadeniz, Salvador Romero-Reyes, Juan Carlos Espín, Ebru Pelvan, Antonio González-Sarrías

**Affiliations:** 1 Laboratory of Food and Health, Research Group on Quality, Safety and Bioactivity of Plant Foods, Department of Food Science and Technology, CEBAS-CSIC, Campus de Espinardo, P.O. Box 164, 30100 Murcia, Spain; mavila@cebas.csic.es (M.Á.Á.-G.); jgbastida@cebas.csic.es (J.A.G.-B.); s.romero@csic.es (S.R.-R.); jcespin@cebas.csic.es (J.C.E.); 2 Life Sciences, TÜBİTAK Marmara Research Center, P.O. Box 21, 41470 Gebze-Kocaeli, Türkiye; bulent.karadeniz@tubitak.gov.tr (B.K.); ebru.pelvan@tubitak.gov.tr (E.P.)

**Keywords:** *Echinacea* extract, INFOGEST, CCD-18Co, chicoric acid, IL-1β, inflammation, polyphenols, hydroxycinnamic acids

## Abstract

*Echinacea purpurea* L. (EP) preparations are globally popular herbal supplements known for their medicinal benefits, including anti-inflammatory activities, partly related to their phenolic composition. However, regarding their use for the management of inflammation-related intestinal diseases, the knowledge about the fate of orally ingested constituents throughout the human gastrointestinal tract and the exposition of in vitro digested extracts in relevant inflammatory models are unknown. This study investigated for the first time the impact of in vitro gastrointestinal digestion (INFOGEST) on the phenolic composition and anti-inflammatory properties of EP extracts from flowers (EF), leaves (EL), and roots (ER) on IL-1β-treated human colon-derived CCD-18Co cells. Among the seven hydroxycinnamic acids identified using HPLC-UV-MS/MS, chicoric and caftaric acids showed the highest concentrations in EL, followed by EF and ER, and all extracts exerted significant reductions in IL-6, IL-8, and PGE_2_ levels. After digestion, despite reducing the bioaccessibility of their phenolics, the anti-inflammatory effects were preserved for digested EL and, to a lesser extent, for EF, but not for digested ER. The lower phenolic content in digested EF and ER could explain these findings. Overall, this study emphasizes the potential of EP in alleviating intestinal inflammatory conditions and related disorders.

## 1. Introduction

Maintaining intestinal homeostasis is crucial for nutrient (re)absorption of water and ions and host defense against exogenous stimuli, maintaining host health [1,2]. Therefore, the inflammatory balance dysfunction in the environment of the intestinal tract, characterized by uncontrolled pro-inflammatory mediators (i.e., IL-6, IL-8, prostaglandins, TNF-α, etc.) and oxidative stress, is involved in most inflammation-related intestinal diseases such as inflammatory bowel diseases (IBDs), including ulcerative colitis and Crohn’s disease, or even colorectal cancer [3,4]. In this line, inflammatory-intestinal diseases are a leading global cause of mortality and morbidity (due to their high incidence and devastating clinical symptoms), remaining a global burden with rapidly increasing incidence and prevalence in developing and industrialized countries [5,6]. Consequently, for decades, there has been growing interest in identifying novel and effective natural molecules from plant-derived extracts that could alleviate intestinal inflammation with fewer side effects than current anti-inflammatory drugs (i.e., aminosalicylates, corticosteroids, immunosuppressive agents, biologics, etc.) [7,8,9].

*Echinacea purpurea* L. (EP) is a perennial herbaceous flowering plant commonly known as purple coneflower. It is the best known of the dozen species of the *Echinacea* genus (*Asteraceae* family) widely cultivated in North America and Europe. EP has been traditionally consumed in different forms for its therapeutic benefits, and therefore, they are among the most popular herbal supplements available worldwide [10,11]. Currently, a variety of extracts and whole-plant EP products, such as direct pressed juices, tea, freeze-dried ethanolic or hydrophilic extracts, and powdered dried in tablet or capsule form, are extensively employed for the prevention or treatment of inflammatory and gastrointestinal diseases, among others [11,12,13]. These benefits have been attributed to different classes of secondary metabolites, such as phenolics, alkylamides, sesquiterpenes, and polysaccharide/glycoproteins [14,15], although the underlying mechanisms remain unclear. Among the phenolics in EP, the main bioactives are caffeic acid derivatives, including chicoric, caftaric, and chlorogenic acids, which exhibit anti-oxidative and anti-inflammatory properties [14,15]. However, the content and type of these bioactive compounds, and particularly phenolics, can vary considerably between different parts of this plant (leaves, flowers, roots, or stem), as well as the environmental and agronomic variables (growth conditions and harvest period) and processing-related factors (extraction procedures, storage conditions, etc.) [10,16]. Thus, despite several preclinical studies reporting anti-inflammatory activities for EP extracts derived from different parts or components of the plant and subjected to various extraction processes [17,18,19,20], there are no studies that have evaluated differences in the efficacy from the different parts of EP collected at the same time and subjected to same extraction process. Regarding intestinal-inflammatory diseases, to the best of our knowledge, only two in vivo studies have reported that the EP extracts exerted a potential protective effect by decreasing inflammation and oxidative stress in ulcerative colitis rat models [21,22]. Similarly, the anti-inflammatory activities of EP extracts and some of their main components, such as chicoric acid, have been mainly investigated in human- and mouse-derived immune cells and other systemic inflammatory models. These studies are associated with their ability to reduce the pro-inflammatory mediators production such as cytokines, reactive oxygen species (ROS), nitric oxide, TNF-α, and IL-1β [14,23,24,25,26]. However, no studies have been conducted on intestinal inflammatory models. Furthermore, little is known about the effectiveness of EP extracts after gastrointestinal digestion.

During digestion, the structures of bioactive compounds, mainly the phenolics, can be hydrolyzed and modified, altering their bioavailability and biological activity (attributable to their absorbable derived metabolites). Even so, native polyphenols and other components could play a crucial role in conferring health benefits in the gastrointestinal tract [27,28]. However, it is mandatory to consider the influence of factors such as gastrointestinal pH, digestive enzymes, electrolyte composition, and bile salts on plant extracts or foods, which modify both their bioaccessibility and bioactivity [29,30]. In this regard, in vitro digestion mimicking the conditions in each stage of gastrointestinal digestion is a helpful method to determine these changes. Despite the large number of models available (presenting physiological differences), in recent years, an international protocol (INFOGEST) has been developed to standardize static in vitro simulated gastrointestinal digestion of foods and nutraceuticals [31].

The aim of the present study was (i) to analyze and compare the phenolic profile of three different EP extract parts (leaves, flowers, and roots) and evaluate the changes under simulated in vitro gastrointestinal digestion (INFOGEST) and (ii) to test their anti-inflammatory effects in an in vitro model of human colon cells. The changes in the phenolic profile, particularly the proportion of chicoric acid, and the anti-inflammatory effects were analyzed to help reveal the post-digestion anti-inflammatory potential of EP extracts. This study is unprecedented as it is the first to explore the anti-inflammatory effects at the intestinal level of the three EP plant parts, with different phenolic concentrations and profiles, under simulated gastrointestinal digestion. Furthermore, this study also provides the scientific basis for highlighting the effective bioactivity of EP preparations in managing inflammation-related intestinal diseases.

## 2. Results

### 2.1. Phenolic Profile in the Echinacea purpurea Plant Parts Extracts

**Figure 1:**
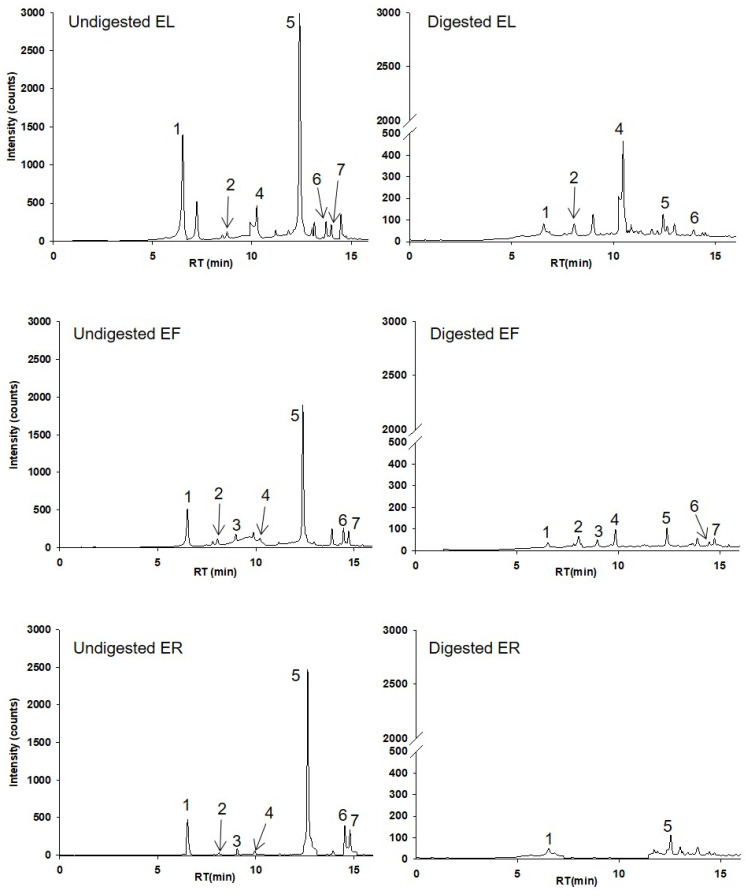
HPLC–UV chromatograms of EP leaves (EL), flowers (EF), and roots (ER) extracts before (undigested) and after digestion (digested) at 320 nm. Chromatographic conditions are described in Section 4.4. The peak identifications are given in Table 1.

As depicted in Table 1, seven phenolics, specifically hydroxycinnamic acids, were identified according to their retention time, mass and mass fragmentation, and UV-Vis characteristics. Chicoric and caffeic acids were identified and quantified from the seven compounds using their authentic standards. Representative chromatograms at 320 nm from EP leaves, flowers, and roots before digestion (undigested) and after digestion (digested) are shown in Figure 1. Peak 1 exhibits the deprotonated molecular ion at *m*/*z*- 311, with a base peak at *m*/*z*- 149 (corresponding to tartaric acid) and a fragment ion at *m*/*z*- 135 (Table 1; Figure 1). Concerning the mono-caffeoylquinic acids (peaks 2 and 3), we tentatively identified peak 2 as chlorogenic acid and peak 3 as neochlorogenic acid based on their relative abundances of the product ions. This determination was made considering that the major product ion at *m*/*z*- 191 corresponds to chlorogenic acid, while *m*/*z*- 179 corresponds to neochlorogenic acid. Peak 4 and peak 5 were quantified as caffeic acid and chicoric acid, respectively, with their authentic standards. Finally, the fragment ions at *m*/*z*- 325 ([M-H-162 (caffeoyl)]-) and 293 [M-H-176 (feruloyl)-18 (H_2_O)]-) allowed to identify tentatively peaks 6 and 7 as feruloylcaffeoyltartaric acids (Table 1; Figure 1).

### 2.2. Quantification and Bioaccessibility of Phenolics in Echinacea purpurea Parts Extracts before and after Digestion

Regarding the quantification of the HPLC analysis of phenolics, as expected in the undigested samples, the most abundant compound was chicoric acid (peak 5; Figure 1), followed by caftaric acid (peak 1; Figure 1), in both cases with the highest concentrations observed in EL compared with EF and ER (*p* < 0.05) (Table 2). There were a few other minor peaks identified and quantified as chlorogenic acid (peak 2; Figure 1), neochlorogenic acid (peak 3; Figure 1), caffeic acid (peak 4; Figure 1), and two feruloylcaffeoyltartaric acid isomers (peaks 6 and 7; Figure 1). These minor compounds also showed different concentrations between the EP parts extracts. While caffeic acid reached 0.31 ± 0.06 mg/g in the EL, the concentrations were much lower in EF and ER (0.03 ± 0.005 and 0.07 ± 0.004 mg/g, respectively) (*p* < 0.05) (Table 2). Noteworthy, chlorogenic acid displayed a higher concentration in EL (0.07 ± 0.009 mg/g) compared to both EF (0.04 ± 0.02 mg/g) and ER (0.03 ± 0.001 mg/g) but were not statistically significant (Table 2). In contrast, the highest concentration of neochlorogenic acid was found in ER (0.06 ± 0.01 mg/g) and undetected in EL but it was not statistically significant (Table 2).

When exposed to simulated gastrointestinal digestion using the INFOGEST methodology, the phenolics detected in ER were highly unstable, detecting only caffeic acid and caftaric acid after digestion. However, the bioaccessibility of caftaric acid was higher in ER compared to EF and EL (Table 2). In this study, the main hydroxycinnamic acids in EP plant parts extracts, chicoric and caftaric acids, showed bioaccessibilities from 3 to 10% and 10 to 39%, respectively. Both chicoric and caftaric acids exhibited lower bioaccessibility rates compared to the rest of the hydroxycinnamic acids. Considering they share tartaric and caffeic acid in their structure, these common elements could contribute to the observed differences with other compounds.

Surprisingly, caffeic acid in EL exhibited a bioaccessibility ratio of 663%. This could be explained by the higher amounts of caftaric and chlorogenic acids in undigested EL samples, as the conversion from caftaric acid might lead to the loss of tartaric acid, and from chlorogenic acid, the loss of quinic acid, potentially resulting in caffeic acid, respectively. All other compound variations are within the experimental error; thus, bioaccessibility can be considered constant.

### 2.3. Effects on Cell Viability

The inflammatory model was run using sub-confluent CCD-18Co cells, which were incubated with non-cytotoxic doses (0.5% in culture medium) of digested and undigested EP extracts. The selected concentrations afforded pH values of 7 and osmolality values of 283–304 mmol/Kg, which were within the tolerance limits of this human cell model. Since chicoric acid was the major phenolic detected in all EP extracts, its anti-inflammatory effect was evaluated in parallel. Two different non-cytotoxic concentrations were assayed: a dose that could be detected in the digested samples at 100% (20 Μm) and another one closer to values tested in samples, considering that they were used at 0.5% (0.5 Μm). In all treatments using this dose, including the blank of digestion (digestion run without EP extracts), cell viability was always above 90% of cell proliferation compared to untreated cells.

### 2.4. Effect on IL-1β-Induced IL-6 and IL-8 Production

**Figure 2:**
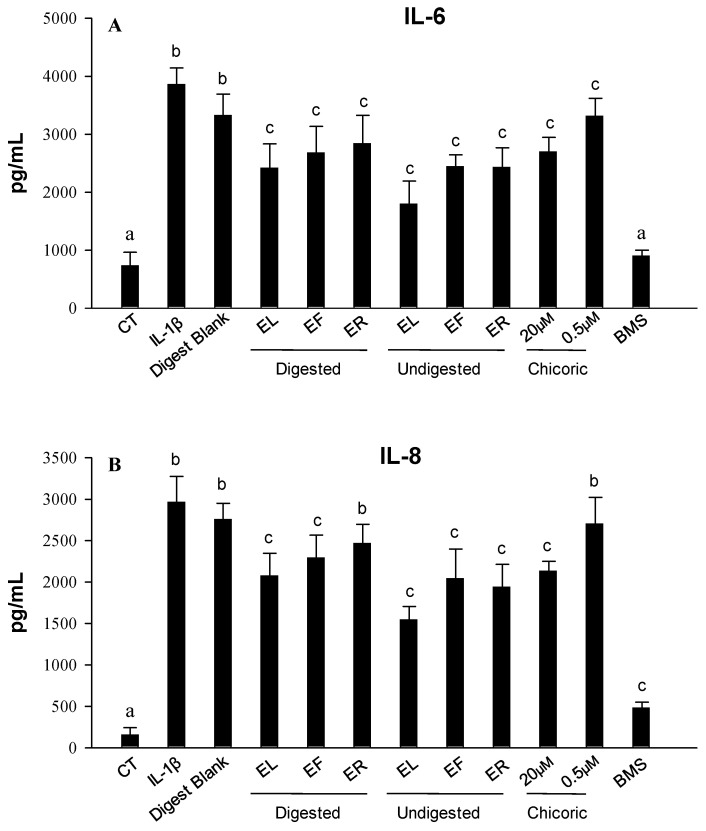
Levels of IL-6 (**A**) and IL-8 (**B**) produced in the CCD-18Co culture media after 18 h of treatment as measured by ELISA after exposure to IL-1β (1 ng/mL) alone or in combination with undigested and digested EP leaves (EL), flowers (EF) and roots (ER) extracts as well as with chicoric acid at 0.5 and 20 μM (0.5% DMSO *v*/*v*). The selective IKK-2 inhibitor (BMS 345541; BMS) at 5 μM was assayed as a positive control of the anti-inflammatory effect. Results are shown as the mean ± SD of three independent experiments. Different letters indicate significant differences *p* < 0.05.

The effects of undigested and digested samples of each EP plant part extract on IL-1β-induced IL-6 and IL-8 production in sub-confluent CCD-18Co myofibroblasts were next investigated. The exposure of the cells to IL-1β led to an increase (*p* < 0.05) in the release of both cytokines compared to both untreated (CT) cells, while no differences were observed with co-treated cells with the digest blank (Figure 2). The inflamed cells co-treated with digested extracts at a non-cytotoxic dose of each EP plant part extract showed a reduction (*p* < 0.05) in the concentration of IL-6 and IL-8 in the medium. However, a lower reduction, but still significant (*p* < 0.05), was observed for IL-6 in undigested samples (Figure 2A). Regarding IL-8 values, lower values, but statistically significant, were detected for digested EL and EF but not for ER samples compared to the undigested samples (Figure 2B). Finally, chicoric acid showed a significant effect in reducing IL-6 levels (*p* < 0.05) compared with IL-1β-induced cells after treatment with both doses, observing a dose-dependence trend but not statistically significant (Figure 2A). Still, only a significant decrease was observed with the treatment of the highest dose tested for IL-8 (*p* < 0.05) (Figure 2B).

### 2.5. Effect on IL-1β-Induced Prostaglandin E2 Production

**Figure 3:**
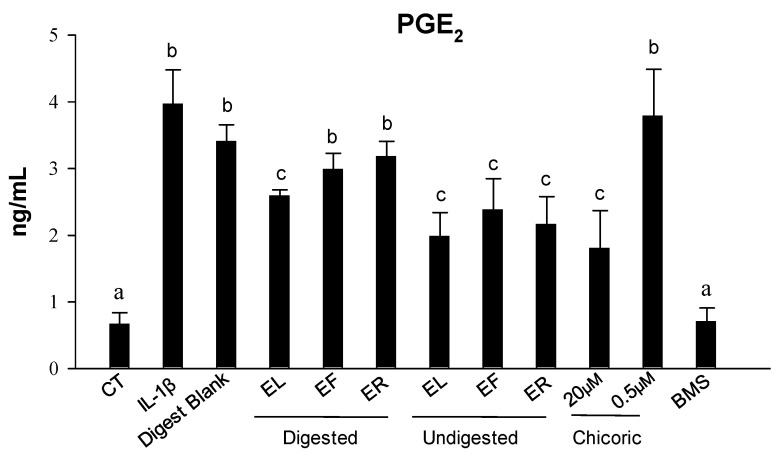
PGE_2_ production levels produced in the CCD-18Co culture media after 18 h of treatment as measured by ELISA after exposure to IL-1β (1 ng/mL) alone or in combination with undigested and digested EP leaves (EL), flowers (EF), and roots (ER) extracts as well as with chicoric acid at 0.5 and 20 μM (0.5% DMSO *v*/*v*). The selective IKK-2 inhibitor (BMS 345541; BMS) at 5 μM was assayed as a positive control of the anti-inflammatory effect. Results are shown as the mean ± SD of three independent experiments. Different letters indicate significant differences *p* < 0.05.

To test whether the undigested and (or) digested EP extracts could modulate PGE_2_ synthesis, we measured its production by CCD-18Co cells stimulated with 1 ng/mL IL-1β for 18 h. Stimulation with IL-1β produced a significant increase (6-fold) in PGE_2_ levels (*p* < 0.05) compared with control cells, while no differences were observed with co-treated cells with the digest blank (Figure 3). Co-treatment with undigested samples (at 0.5%) significantly (*p* < 0.05) decreased PGE_2_ levels (around 3-fold for each condition). However, only the digested EL extract significantly decreased PGE_2_ levels (*p* < 0.05) but not the digested ER and EF samples. Additionally, the treatment with chicoric acid only showed a significant effect (*p* < 0.05) at the highest dose tested (20 μM) (Figure 3).

### 2.6. Principal Components Analysis (PCA) for the Anti-Inflammatory Responses of EP Plant Parts before and after Digestion

**Figure 4:**
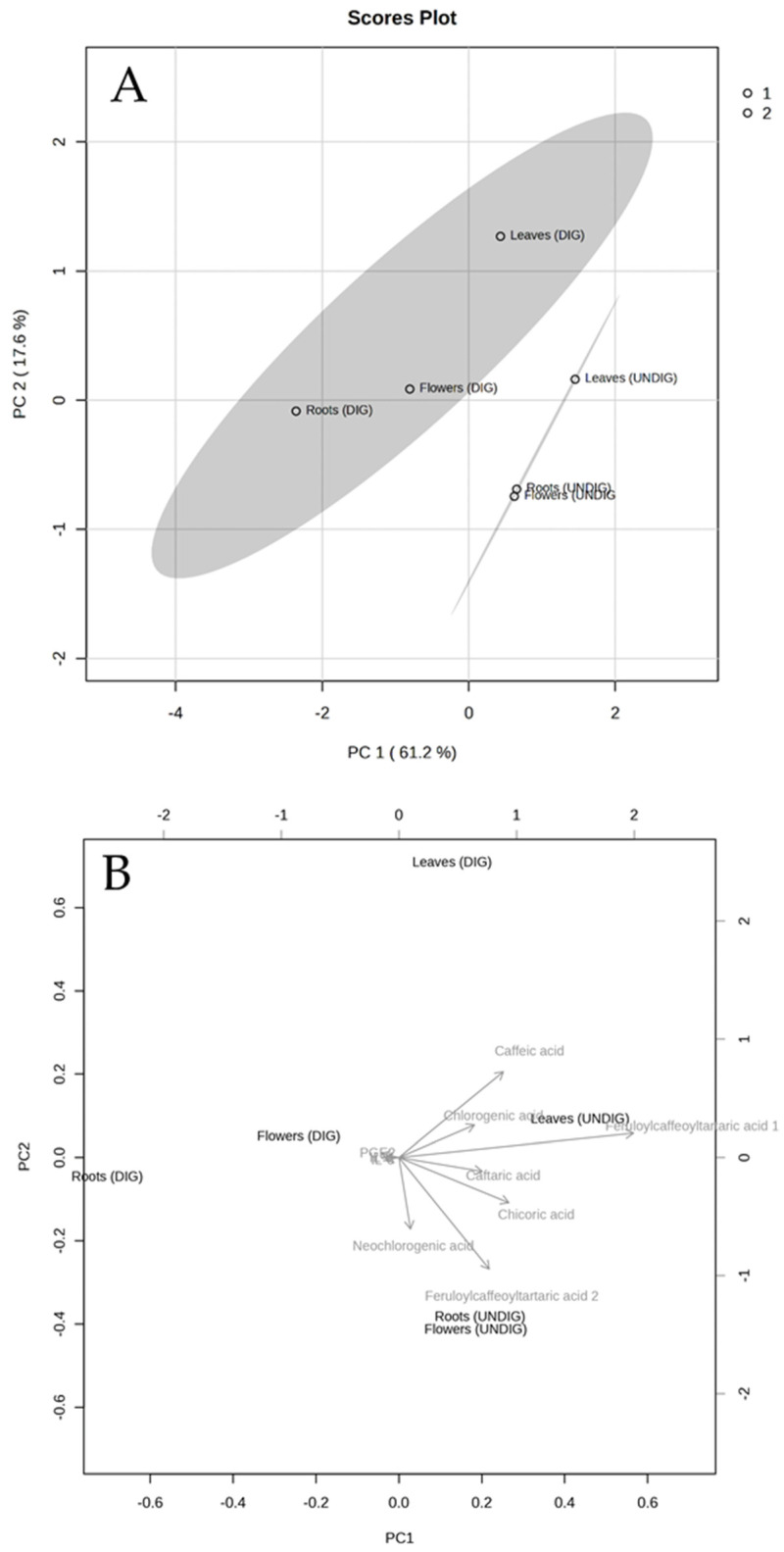
Principal component analysis (PCA) (**A**) of the EP leaves, flowers, and root extracts before (UNDIG) and after digestion (DIG) (phenolic compounds and pro-inflammatory markers). The biplot (**B**) displays the interplay between EP samples and phenolics and pro-inflammatory markers (denoted by arrows) in a two-dimensional space.

A principal component analysis (PCA) was applied to determine the relationship between the phenolics detected in each EP extract before and after digestion and the three pro-inflammatory markers analyzed (IL-6, IL-8, and PGE2). Figure 4 shows the PCA analysis of the evaluated responses in CCD18-Co cells after treatments. The PCA performed on the data matrix that included the concentration of each phenolic detected (in each plant part before and after digestion) in the separated matrices, as well as the concentration of pro-inflammatory markers (IL-6, IL-8, and PGE2) after treatment with each sample revealed that the two principal components (PC1 and PC2) explained 61.2% and 17.6% of the variance, respectively (Figure 4A). In addition, PCA showed the clustering into two main groups, according to the concentration of the phenolic detected (see Table 2), the three EP extracts undigested (UNDIG; non-colored) and digested (DIG; colored). Furthermore, among each group, both EL extracts (undigested and digested ones) were located separately from the other EP plant part extracts (EF and ER).

On the other hand, the joint analysis of the biplot results confirmed that the three pro-inflammatory mediators evaluated correlated negatively (indicated by arrows in opposite directions) with the presence of all phenolics detected in the EP samples (Figure 4B), indicating the potential role between the concentration of bioactive EP phenolics and their capacity to protect against inflammation in CCD18-Co cells.

## 3. Discussion

Chronic inflammation of the gastrointestinal tract is characterized by increased levels of reactive oxygen species (ROS) and pro-inflammatory cytokines in the intestinal mucosa, causing tissue damage as part of the progression of many chronic diseases such as IBDs or colorectal cancer [3,32]. The incidence of these diseases continues to increase globally, and over 2.5 million people in the USA and 10 million in Europe are estimated to have IBD, with substantial costs for health care [5,6]. In this regard, there has also been increasing research interest in extracting, purifying, and identifying natural compounds and extracts with lower side effects than existing drugs for preventing or treating intestinal diseases. Likewise, many current studies have revealed that the benefits of polyphenols present in herbal preparations and plant foods are linked with intestinal health, including anti-oxidative stress and anti-inflammation [2].

Different preparations containing EP, used as a medicinal plant since the end of the last century, alone and less frequently mixed with other EP species (*E. angustifolia* and *E. pallida*), are among the top-selling herbs in Europe and the United States. They are widely used to treat inflammation and gastrointestinal diseases due to the presence of both phenolic compounds (caffeic acid derivatives, chlorogenic acid, and chicoric acid) and other compounds such as alkylamides and glycoproteins [11,17]. However, it has been identified that the location and content of these constituents, mainly polyphenols, which exhibit promising antioxidants and anti-inflammatory activities, change over time and vary between plant parts [33,34]. Apart from the extraction procedure, other factors, including climate, harvest period, processing, and storage conditions, influence a substantial variability in the bioactive components [35,36,37]. Thus, we hypothesize that the parts of the EP with different phenolic profiles may differ in their anti-inflammatory activity and, therefore, their comparison combined with their characterization could help identify the components responsible for the biological activity. In this study, seven phenolics were identified, with chicoric acid being the most abundant compound, followed by caftaric acid, in agreement with previous information found in the literature [37,38,39]. The content of these compounds showed differences between the different EP plant parts extracts, showing higher concentrations in EL compared to ER and EF. Previous studies have reported a richer phenolic profile in EL extracts [34,37] compared with EF [40] and ER [41], which is consistent with our results. However, we have not detected other minor compounds, like other hydroxycinnamic acids such as echinacoside and cynarin, or flavonols such as kaempferol derivatives, which have also been identified in Echinacea species [34,37,42]. As detailed above, the presence or absence of these constituents could depend on multiple factors, including the extraction process used in the preparation [37,43,44]. In the present study, we performed an HPLC analysis employing an extraction method previously reported for various phenolics in plant extracts [45,46]. Although some research compares extraction with different solvents (e.g., ethanol, acetone, or methanol) [37,39], to our knowledge, there are no studies that reflect a singular method for analyzing different compounds in various EP parts.

On the other hand, under some conditions, (poly)phenols can be unstable and usually degrade during digestion, which could affect their potential health benefits [27]. Thus, the present study highlights a fundamental issue concerning the need to investigate their bioaccessibility using a standardized protocol to simulate digestion. As shown in this study, the exposure of these extracts to in vitro digestion allowed us to identify a decrease in the concentration of phenolics in all EP extracts, showing bioaccessibility ratios of chicoric and caftaric acids from 3 to 10% and from 10 to 39%, respectively. Previous reports have described a similar % of bioaccessibility for other hydroxycinnamic acids [34]. However, to the best of our knowledge, this is the first study reporting the in vitro gastrointestinal stability of EP. Despite the low phenolic concentration observed after digestion, statistically significant anti-inflammatory activity was still observed, although less than that observed for their corresponding extracts not subjected to in vitro digestion. This methodology is a useful assay to determine the changes and bioaccessibility of phenolic compounds in the matrix of each EP extract concerning the initial total content, which would be accessible both for intestinal absorption and to exert a beneficial activity in the gastrointestinal tract. Nevertheless, the present study overlooks the possible role of the gut microbiota in the complex transformations of EP phenolics. Further studies are warranted to explore this crucial aspect.

Overall, digestion significantly reduced the effects of EL, EF, and (or) ER extracts on IL-1β-induced IL-6, IL-8, and PGE_2_ production in sub-confluent CCD-18Co myofibroblasts compared to the undigested extracts, except for digested ER (for IL-8) and digested ER and EF (for IL-8 and PGE_2_). These results indicate, for the first time, the potential of EP preparations after digestion to alleviate intestinal inflammation. However, the mechanism through which EP extracts exert anti-inflammatory activity is still unclear. These results agree with previous in vitro studies conducted with different EP extracts on systemic inflammatory models [14,23,24,25,26], as well as few in vivo studies that reported anti-inflammatory activity (reducing IL-6, PGE_2,_ and oxidative stress level, among others) in acetic acid-induced ulcerative colitis in rats [21], potassium dichromate-induced nephrotoxicity in rats [20], and in renal tissues of LPS-treated mice [47].

Furthermore, both digested and undigested EL extracts exerted the highest anti-inflammatory effect for the markers assayed, which could be explained, at least partly, by the higher phenolic content, mainly chicoric acid, compared with the other plant part extracts (EF and ER). Thus, in the present study, a PCA analysis corroborated that the three pro-inflammatory mediators evaluated showed a negative correlation with the presence of all phenolics detected in the EP extracts. In this line, in the present study, chicoric acid showed an anti-inflammatory effect at concentrations attainable after the in vitro digestion, suggesting that phenolics could be partly related to the anti-inflammatory effects. Other preclinical studies have reported that chicoric acid and (or) caffeic acid can exert anti-inflammatory activities [48,49,50,51]. However, in other studies, the anti-inflammatory properties have been attributed to other EP components such as alkylamides, sesquiterpenes, and (or) polysaccharides [14,52,53]. In this regard, the aerial parts of EP, apart from phenolics, contain a high content of polysaccharides, glycoproteins, and alkylamides [34]. Therefore, we cannot discard that the anti-inflammatory activity could arise from the synergy between the different bioactives in the EP plant part extracts.

## 4. Materials and Methods

### 4.1. Reagents

HPLC-grade acetonitrile (ACN), formic acid, dimethyl sulfoxide (DMSO), and methanol (99.8% LC–MS; MeOH) were obtained from J.T. Baker (Deventer, The Netherlands). Ethanol (≥99.8%) was purchased from Merck (Darmstadt, Germany). Unless stated otherwise, all other reagents were purchased from Sigma-Aldrich (St. Louis, MO, USA). Ultrapure Millipore water was used throughout the study.

### 4.2. Plant Materials and Extract Preparation

Purple coneflower (*Echinacea purpurea* L.; EP) was obtained from a local company (Nativital Doğal Yaşam ve Sağlık Ürünleri San. Tic. Ltd. Şti., İkitelli Osb/Başakşehir/İstanbul, Türkiye), where the purple coneflower grown in Bandırma/Balıkesir region with the latitude and longitude 40°21′17″ North 27°58′11″ East. Different parts (flowers, leaves, roots) of EP were separated manually, dried (roots were dried under the sun while other parts dried under shade), and subsequently extracted as mentioned in Turkish Pharmacopeia (with slight modifications) [54] with 70% ethanol:water mixture (1:10, *v*/*v*) for 1 h at 40 °C by shaking before ethanol evaporation in the filtered supernatant. The aqueous part was freeze-dried (Christ Epsilon 2–4 Lyo-Screen-Control-LSC, Osterode am Harz, Germany) to obtain a powder used for further studies.

### 4.3. Static In Vitro Gastrointestinal Digestion

The in vitro gastrointestinal digestions were performed in 3 phases (oral, gastric, and intestinal) by preparing specific simulated digestive fluids of salts and digestive enzymes and adjusting the temperature and pH following the standardized INFOGEST protocol [31,55] with minor modifications. Briefly, 300 mg of each extract reconstituted in water (10% *w*/*v*) in separate 50 mL tubes were mixed with 5 mL of simulated salivary fluid, including α-amylase (1500 U/mL) for 2 min at pH 7.0. The mixture was then diluted using 10 mL of simulated gastric fluid containing pepsin (25,000 U/mL). The sample was incubated at 37 °C for 2 h, at pH 3.0. At the end of this stage, the pH was adjusted to pH 7.0, and 20 mL of simulated intestinal fluid and pancreatin (800 U/mL) were added to this mixture and incubated at 37 °C for 120 min. At the end of the intestinal phase, all samples were centrifuged at 5000 rpm for 10 min. The supernatants (soluble, bioaccessible fraction) were kept at −80 °C until the (poly)phenolic profile and anti-inflammatory effects were evaluated. Additionally, a digest blank was run in parallel without any EP extract (replaced by water) that was used as the negative control. The in vitro gastrointestinal digestions were performed in three independent replications, and pooled samples were used for the subsequent assays.

### 4.4. Polyphenolic Content Analysis of Echinacea purpurea Extracts before and after Digestion

EP-powered roots, flowers, and leaves extracts (EL, EF, and ER) (50 mg each) were re-suspended with 10 mL MeOH/DMSO/H_2_O (40:40:20, *v*/*v*/*v*) containing 0.1% HCl (*v*/*v*). The samples were vortexed for 2 min, sonicated in an ultrasonic bath for 5 min, and centrifuged at 4000× *g* for 5 min at room temperature. The process was repeated using a 5 mL extraction solution to obtain the highest quantity of phenolic compounds. The supernatant was collected and filtered using a 0.45 µm polyvinylidene difluoride (PVDF) filter before analysis using HPLC-MS/MS. Three replicates were extracted and analyzed for each sample.

Gastrointestinal digestion samples were thawed and analyzed as reported elsewhere with some modifications [56]. Briefly, samples from the undigested and digested extracts were extracted with a pure MeOH solution in a 1:1 (*v*/*v*) proportion to each tube to precipitate the remaining enzymes. Samples were homogenized using vortex for 2 min, centrifuged at 4 °C for 15 min at 10,000× *g*, and the supernatant was recovered. Next, each sample was evaporated in a speed vacuum and re-suspended in 100 µL MeOH. Final samples in MeOH were filtered using 0.45 µm PVDF filters and added to a vial before HPLC analysis. Each digested sample was analyzed in triplicate. The stability of the phenolic compounds was evaluated in the intestinal phase. Bioaccessibility was calculated as the ratio between the final concentration at the end of the digestion (intestinal fraction) and the initial concentration in the undigested samples [57].

The matrix effect (%ME) was calculated for chicoric acid and caffeic acid by comparing the slopes of their calibration curves in the solvent (MeOH) with those obtained by spiking blank samples from the gastrointestinal digestion with known concentrations of chicoric acid and caffeic acid, (post-extraction spiked sample): %ME = ((slope of the calibration curve in the matrix − slope of the calibration curve in MeOH)/slope of the calibration curve in MeOH) × 100. As described elsewhere [58], we set a ratio of ±15% to determine the influence or absence of ME. For ratio values >15% (i.e., chicoric and caffeic acids), calibration curves were prepared in the matrix to quantify the digested EP plant parts extracts.

The determination of phenolic compounds was performed by HPLC (Agilent 1200 system-coupled to diode array (DAD) and ion trap (IT) mass spectrometer detectors; Santa Clara, CA, USA) analysis using extraction methods previously reported for various phenolics in plant extracts [45]. Compound identification was based on elution order, UV spectra, molecular weight, and MS/MS fragmentation. Hydroxycinnamic acids were quantified using chicoric acid as a standard at a wavelength of 320 nm.

### 4.5. Cell Culture

As a cellular model, we used the colon CCD-18Co myofibroblast cell line (ATCC^®^ CRL-1459) from the American Type Culture Collection (ATCC, Rockville, MD, USA). According to the ATCC recommendations, the culture medium selected was EMEM (pH 7.2–7.4) enriched with FBS (10% *v*/*v*) and supplemented with antibiotics (streptomycin and penicillin at 100 mg/mL and 100 U/mL, respectively), 1.5 g/L sodium bicarbonate, 1 mM sodium pyruvate, 0.1 mM nonessential amino acids, and 2 mM L-glutamine. The growth and maintenance of the cells were carried out as follows: cells seeded at 6000 cells/cm^2^ in T75-flasks incubated at 37 °C in incubators set with optimum growth conditions (constant humidity and 5% CO_2_/95% air atmosphere) for 4–5 days. At confluences ≥ 80%, the cells were subcultured and seeded at concentrations optimized for maintenance or treatments. The population doubling levels (PDL) range used in all experiments was from 26 to 32.

### 4.6. Cell Viability and Inflammatory Assay

First, the maximum concentration of undigested and digested EP extracts (5, 2.5, 1, and 0.5%) lacking cytotoxic effects on colon myofibroblasts was tested. Culture medium osmolarity and pH were measured using a vapor pressure osmometer 5520 (VAPRO Wescor, Logan, UT, USA) and a pH indicator paper (Neutralit, pH 5.5–9.0, Merck) inside the incubator. Chicoric acid, solubilized in DMSO and sterilized (filtered by 0.22 μm), was tested at dosages of 0.5 and 20 μM (<0.5% DMSO in the culture medium). Once these parameters were optimized, the cells were seeded at 10,000 cells/well in 96-well plates and incubated for 2 days. The attached cells were then incubated in EMEM supplemented with 0.1% FBS (*v*/*v*) for 24 h. Next, sub-confluent myofibroblasts were co-treated with 1 ng/mL IL-1β and with the filter sterilized (0.22 μm) undigested/digested extracts and digest blank samples at non-cytotoxic concentrations (0.5%) for 16 h. Unstimulated cells were used as the negative control. The selective IKK-2 inhibitor (BMS 345541; BMS) at 5 μM was assayed as a positive control of the anti-inflammatory effect. After the inflammatory assay, the culture medium was collected and frozen at −80 °C for further analysis. The CCD-18Co cell viability and proliferation were measured using the MTT reduction assay at 24 h, as previously described [46,59], to confirm that the treatments with all the samples did not exert an anti-proliferative and (or) cytotoxic effect. The dose of 0.5% yielded similar values to control cells regarding cell viability and proliferation, showing no statistically significant differences. Even so, cell proliferation data obtained by MTT reduction assay after each treatment were used to normalize the values of the inflammatory markers. Assays were repeated three times (n = 3). The treatments were tested in 6–8 different wells in each assay.

### 4.7. Effect of the Echinacea purpurea Extracts on PGE2 and Cytokines Production in IL-1β-Stimulated Myofibroblasts

The PGE2 content (ELISA kit from Cayman, San Diego, CA, USA) and the pro-inflammatory cytokines IL-8 and IL-6 (ELISA kits from PeproTech, Rocky Hill, NJ, USA) were analyzed in the culture medium using an absorbance-detecting microplate reader (Infinite M1000 Pro, Tecan, Grodig, Austria). The data were expressed as average ± SD from three independent assays (n = 3). The culture medium of the different treatments (carried out in each replicate) was pooled from six to eight different wells.

### 4.8. Statistical Analysis

Data were expressed as the average ± standard deviation (SD). A two-tailed unpaired Student’s *t*-test was used for statistical analysis of the data using SPSS Software, version 27.0 (SPSS Inc., Chicago, IL, USA). Multiple comparisons were performed using Graph Pad Prism 9.0 (GraphPad Software, San Diego, CA, USA) to perform a univariate analysis of variance (ANOVA) with Tukey’s post-hoc test. *p* < 0.05 was used to determine whether the differences were significant. Data sets obtained from the HPLC-UV-MS/MS analysis for each EP sample and the three pro-inflammatory markers evaluated were imported into MetaboAnalyst 6.0 for a principal component analysis (PCA) to examine the interrelationships between phenolic profiles and anti-inflammatory effect. Graphs were performed using Sigma Plot 14.5 (Systat Software, San Jose, CA, USA). A value of *p* < 0.05 was considered to be statistically significant.

## 5. Conclusions

This is the first time that the bioaccessibility and anti-inflammatory activity of different EP plant parts have been investigated before and after an in vitro gastrointestinal digestion. Overall, this study contributes to the limited pre-existing knowledge carried out in models with little physiological relevance and, therefore, could be used as a key reference in future studies on the beneficial effects of herbal and natural supplements, mainly at the gastrointestinal level. Thus, the present study has demonstrated that the anti-inflammatory effects of different EP plant part extracts are preserved after in vitro gastrointestinal digestion despite a reduction in the concentration of their phenolics. The comparison of the extracts has shown that EL extracts have a higher phenolic content than EF and ER. Subsequently, EL exerted greater activity. With the caution that in vitro results cannot be directly translated to in vivo conditions, the current study suggests that these EP plant extracts may have a beneficial effect on colonic inflammation and, therefore, the EP products, mainly from leave extracts, but also flower or root, have the potential to be used in food or nutraceutical formulations to prevent IBDs or other chronic inflammatory pathologies. Further investigations on the gut microbiota metabolism of EP extracts and the resulting bioactivity are required in future in vivo studies.

## Figures and Tables

**Table 1:** Characterization of the major compounds found in the different *Echinacea* plant parts extracts.

Peak	RT (min)	Compound	[M-H]^−^ (*m*/*z*)	MS2 (*m*/*z*)
1	6.98	Caftaric acid	311	197/149
2	8.22	Chlorogenic acid	353	311/191
3	9.21	Neochlorogenic acid	353	312/191/179
4	10.09	Caffeic acid ^1^	179	157/135
5	12.66	Chicoric acid ^1^	473	311/293/149
6	14.68	Feruloylcaffeoyltartaric acid 1	487	325/293/285
7	14.93	Feruloylcaffeoyltartaric acid 2	487	325/293/179

Retention time (RT). ^1^ Identified using the authentical standard.

**Table 2:** Digestive stability and bioaccessibility of phenolics found in the different EP plant parts extracts.

Peak	Compound	EL (Undigested)	EL (Digested)	B (%)	EF (Undigested)	EF (Digested)	B (%)	ER (Undigested)	ER (Digested)	B (%)
1	Caftaric acid	2.66 ± 0.17 *^a^	0.28 ± 0.12 ^b^	10 ± 4	0.56 ± 0.08 ^c^	0.13 ± 0.04 ^d^	23 ± 5	0.45 ± 0.02 ^c^	0.18 ± 0.008 ^b,d^	39 ± 2
2	Chlorogenic acid	0.07 ± 0.009	0.07 ± 0.03	98 ± 53	0.04 ± 0.02	0.05 ± 0.02	120 ± 93	0.03 ± 0.001	nd	-
3	Neochlorogenic acid	nd	nd	-	0.03 ± 0.006	0.05 ± 0.008	180 ± 68	0.06 ± 0.01	nd	-
4	Caffeic acid ^1^	0.31 ± 0.06 ^a^	0.32 ± 0.03 ^a,b^	106 ± 13	0.03 ± 0.005 ^c^	0.23 ± 0.01 ^a^	663 ± 65	0.07 ± 0.004 ^c^	nd	-
5	Chicoric acid ^1^	9.54 ± 0.71 ^a^	0.42 ± 0.05 ^b^	4 ± 1	4.85 ± 1.07 ^c^	0.46 ± 0.09 ^b,d^	10 ± 2	7.30 ± 0.51 ^e^	0.26 ± 0.04 ^b,d^	3 ± 0.4
6	Feruloylcaffeoyltartaric acid 1	0.59 ± 0.12 ^a^	0.58 ± 0.05 ^a,b^	102 ± 27	0.28 ± 0.06 ^c^	0.10 ± 0.03 ^d^	36 ± 2	0.43 ± 0.03 ^a,b,c^	nd	-
7	Feruloylcaffeoyltartaric acid 2	0.14 ± 0.02 ^a^	nd	-	0.23 ± 0.05 ^b^	0.07 ± 0.03 ^a^	31 ± 7	0.52 ± 0.03 ^c^	nd	-
**Total phenolics**		**13.3 ± 1.09**	**1.67 ± 0.28**		**6.02 ± 1.28**	**1.08 ± 0.27**		**8.87 ± 0.61**	**0.43 ± 0.04**	

* Values are the mean of three independent determinations ± standard deviation. The quantification (mg/g extract) of the phenolic compounds in leaves, flowers, and root extracts of EP before and after digestion was quantified at 320 nm with chicoric acid.^1^ Quantified with the authentical standard; EP, *Echinacea purpurea* L.; EL, *Echinacea purpurea* leaves extract; EF, *Echinacea purpurea* flowers extract; ER, *Echinacea purpurea* roots extract; B: Bioaccessibility; nd: not detected. Different lowercase letters in the same row indicate significant differences (*p* < 0.05).

## Data Availability

Data are contained within the article.
